# Magnetic resonance imaging changes in a 69-year old man with hypoglycemia induced brain injury: case report and literature review

**DOI:** 10.11604/pamj.2019.32.131.17967

**Published:** 2019-03-20

**Authors:** Pranitha Reddy Arrabyru, Ihtesham Aatif Qureshi, Lauren Skalomenos, Gustavo Jose Rodriguez

**Affiliations:** 1 Neurology Department, Texas Tech University Health Sciences Center, El Paso, Texas, United States

**Keywords:** Hypoglycemia, stroke, MRI, hippocampus, ICU, glucose, focal deficits

## Abstract

We present the case of a 69-year old man who was brought to the hospital after being found unconscious; last seen at baseline 9 hours prior. On admission he was found to be severely hypoglycemic and received prompt glucose administration, with no immediate neurological improvement. Stroke was suspected. A brain MRI revealed abnormal hyperintense signal involving the head and tail of the left hippocampus. After close neurological monitoring and supportive care in the ICU, his condition improved over time, leaving no residual focal deficits. This case highlights the presence of MRI changes in patients with severe hypoglycemia as it happens in hypoglycemic coma.

## Introduction

The clinical presentation of hypoglycemia is variable and while it is influenced by the rate and degree of decrease in blood glucose concentration, prognostic factors depend on the severity and duration [1]. The American Diabetes Association (ADA) describes hypoglycemia as a clinical syndrome characterized by a plasma glucose concentration of less than 70mg/dL (3.9 mmol/L) with or without symptoms [2]. When blood glucose is below 68mg/dL (3.8 mmol/L), neurogenic symptoms like sweating, tachycardia, trembling and hunger can occur due to glucagon and epinephrine release, followed by neuroglycopenic symptoms including seizures, cognitive disturbances, hemiplegia and confusion. When a blood glucose level is <49mg/dL (2.72 mmol/L) patients may become comatose [3]. Persistent vegetative state and death can follow in most severe cases. Symptomatic hypoglycemia, especially when hemiparesis or coma are present can be misdiagnosed as acute ischemic stroke. Both symptomatic hypoglycemia and acute ischemic stroke share, but also differ in clinical, radiological and neurochemistry features [4]. A correct diagnosis is essential in the early stages, since treatments vary for hypoglycemia and acute ischemic stroke. The initial approach should include a glycemic check, as hemiparesis and early coma related to hypoglycemia respond to prompt administration of intravenous glucose improving the neurological symptoms. Herewith, we report a case of drug induced hypoglycemic coma with brain magnetic resonance imaging (MRI) findings that could be misinterpreted as acute ischemic stroke.

## Patient and observation

**Figure 1:**
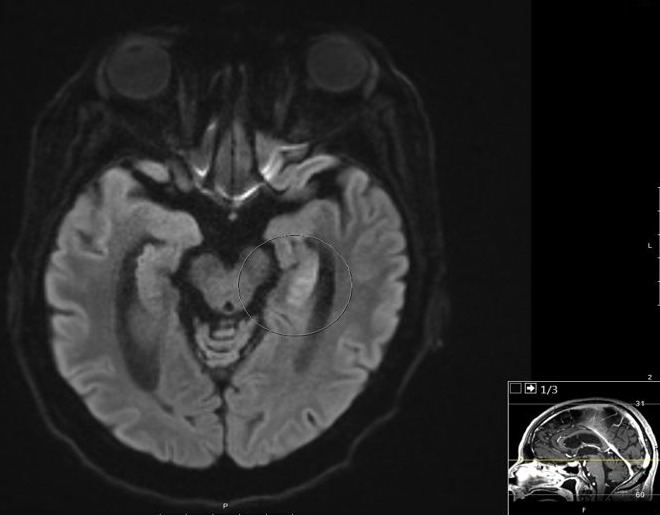
A magnetic resonance imaging (MRI) brain showing an abnormal hyperintense signal involving the head of the left hippocampus on diffusion weighted image (DWI) sequence

**Figure 2:**
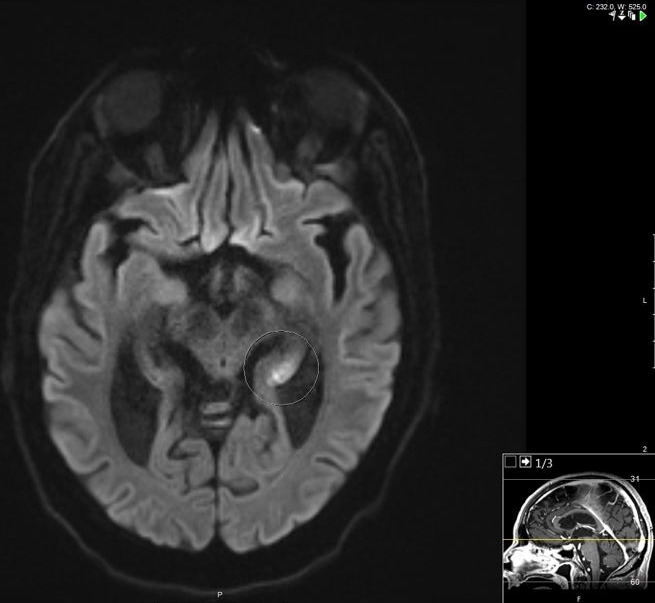
A magnetic resonance imaging (MRI) brain showing an abnormal hyperintense signal involving the tail of the left hippocampus on diffusion weighted image (DWI) sequence

**Figure 3:**
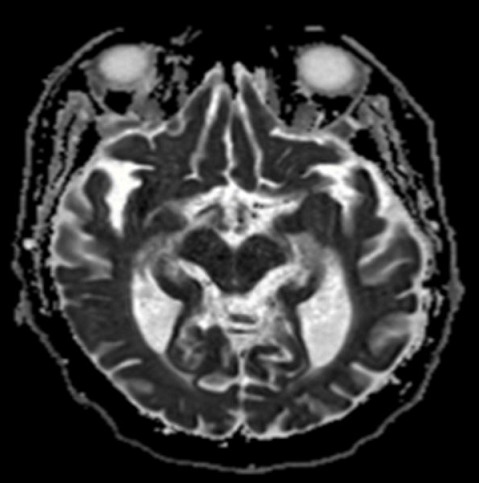
A magnetic resonance imaging (MRI) brain showing a corresponding hypointense signal on apparent diffusion coefficient (ADC) sequence

A 69 year-old-man with a past medical history of hypertension and insulin dependent diabetes was admitted to the hospital after being found unconscious at his home. He was last seen normal about 9 hours prior to the emergency department arrival. Laboratory results revealed hypoglycemia, a blood sugar level of 29 mg/dL (1.6 mmol/L) on arrival. He received intravenous glucose (50% dextrose) immediately as per hypoglycemia protocol. Since his Glasgow Coma Scale (GCS) was low (<8) and with poor airway protection, he underwent endotracheal intubation with mechanical ventilation. There was no immediate clinical improvement even after the serum glucose was normalized. As per the family, he has had several episodes of hypoglycemia in the past and had responded well to immediate replenishment of glucose. Recent dosage modifications of his home insulin by his primary care physician had occurred due to the hypoglycemic events. On neurological examination, he was comatose, not following commands, there was no eye opening to noxious stimuli, pupils appeared to be equal and reactive to light and roving eye movements were noted. Doll's eye phenomenon was preserved. In response to painful stimuli, bilateral flexion of upper extremities was noted. Absent bilateral Babinski's reflex. Stroke was considered in the differential diagnosis. Computed tomography (CT) of head without contrast showed no hemorrhage and CT angiography (CTA) of head and neck was negative for large vessel occlusion. A magnetic resonance imaging (MRI) of brain revealed a hyperintense signal involving the head and tail of the left hippocampus on diffusion weighted image (DWI) sequence (Figure 1, Figure 2). A corresponding hypointense lesion was noted on apparent diffusion coefficient (ADC) sequence (Figure 3). He was transferred to the intensive care unit (ICU) for close neurological monitoring with frequent glycemic checks. His condition improved and was extubated a few days later, he was discharged home with no residual focal deficits.

## Discussion

Hypoglycemic coma due to prolonged low serum glucose levels can be a difficult clinical diagnosis given the lack of prompt response to glycemic replacement. It can clinically and radiologically mimic other conditions including ischemic stroke. The brain requires a constant supply of glucose as it cannot synthesize glucose and only astrocytes store considerable amounts of glycogen. The arterial plasma glucose concentration dictates effective transport of glucose to the brain through the process of facilitated diffusion. If this blood glucose supply is not adequate to meet the metabolic demands of the brain, signs and symptoms of injury ensues. The pathophysiological mechanism of hypoglycemic brain injury is still unclear. One theory suggests that decreased serum glucose promotes cellular energy depletion in neurons and the astrocytes, leading to the failure of membrane ionic pumps, impaired protein synthesis, excessive aspartate production and loss of membrane ion homeostasis, initiating a shift of water from the extracellular space into the intracellular space [5]. This excitotoxic edema appears in diffusion weighted imaging (DWI) as hyperintense signal with a corresponding decreased apparent diffusion coefficient (ADC). Excitotoxic edema related to hypoglycemia is more commonly observed in the cerebral cortex, hippocampus and dorsal striatum and rarely seen in the cerebellum, brain stem or hypothalamus likely due to a higher activity of the glucose transporter [4]. In addition, experimental studies have disclosed higher levels of adenosine triphosphate (ATP) in the thalami, also explaining the absence of thalamic involvement in hypoglycemia [4]. During ischemia, there is lack of both oxygen and glucose leading to the accumulation of glutamate and cell death [6]. Although glucose is the main source of energy for the brain, brain cells can also utilize fructose, glutamate, glutamine and ketone bodies as an emergency fuel supply, because of the presence of oxygen. In hypoglycemia due to series of metabolic events, more of aspartate (less glutamate) is released into the extracellular space leading to a potentially reversible cytotoxic edema, depending on the duration [6, 7]. Our patient was found to have an abnormality in the hippocampal region on DWI/ADC imaging in accordance with the previously discussed proposed mechanism. We noticed a significant neurological improvement followed by a complete recovery with treatment in our patient. This finding is also consistent with the work of Kang *et al*. who reported that hippocampal involvement does not predict a poor outcome in the setting of hypoglycemia [8]. Moreover DWI/ADC lesions in the setting of hypoglycemia can be reversible as previously reported by Aoki *et al.*, Lo *et al.* [5, 9]. Our case lacks follow up neuroimaging, however given the clinical improvement it was thought to be unnecessary. In acute symptomatic hypoglycemia, brain imaging findings are not always diffuse or bilateral. Very few cases reported unilateral involvement and is considered to be rare, as seen in our patient [4]. The possible mechanism for the unilateral involvement of a hypoglycemic lesion in our patient is the presence of metabolic asymmetry. Shen *et al*. explained in his study, the presence of decline in normal aging glucose metabolism in the left hemisphere in comparison to right, thus highlighting the presence of metabolic asymmetry between the left and right hemispheres [10].

## Conclusion

In clinical practice, hypoglycemic coma can be misdiagnosed as stroke, as they share clinical and radiological features. Its prompt diagnosis and treatment can lead to a rapid clinical recovery with complete resolution of imaging findings.

## Competing interests

The authors declare no competing interests.
